# Using the Pareto principle in genome-wide breeding value estimation

**DOI:** 10.1186/1297-9686-43-35

**Published:** 2011-11-01

**Authors:** Xijiang Yu, Theo HE Meuwissen

**Affiliations:** 1 Department of Animal and Aquacultural Sciences, Norwegian University of Life Sciences, 1432 Ås, Norway

## Abstract

Genome-wide breeding value (GWEBV) estimation methods can be classified based on the prior distribution assumptions of marker effects. Genome-wide BLUP methods assume a normal prior distribution for all markers with a constant variance, and are computationally fast. In Bayesian methods, more flexible prior distributions of SNP effects are applied that allow for very large SNP effects although most are small or even zero, but these prior distributions are often also computationally demanding as they rely on Monte Carlo Markov chain sampling. In this study, we adopted the Pareto principle to weight available marker loci, i.e., we consider that *x*% of the loci explain (100 - *x*)% of the total genetic variance. Assuming this principle, it is also possible to define the variances of the prior distribution of the 'big' and 'small' SNP. The relatively few large SNP explain a large proportion of the genetic variance and the majority of the SNP show small effects and explain a minor proportion of the genetic variance. We name this method MixP, where the prior distribution is a mixture of two normal distributions, i.e. one with a big variance and one with a small variance. Simulation results, using a real Norwegian Red cattle pedigree, show that MixP is at least as accurate as the other methods in all studied cases. This method also reduces the hyper-parameters of the prior distribution from 2 (proportion and variance of SNP with big effects) to 1 (proportion of SNP with big effects), assuming the overall genetic variance is known. The mixture of normal distribution prior made it possible to solve the equations iteratively, which greatly reduced computation loads by two orders of magnitude. In the era of marker density reaching million(s) and whole-genome sequence data, MixP provides a computationally feasible Bayesian method of analysis.

## Introduction

Genomic selection (GS) is currently being adopted by the dairy cattle breeding industries around the world [1]. Genome-wide breeding value (GWEBV) prediction plays a pivotal role for this new technology. Its accuracy depends on the statistical methods used, the genome, the population structure, and trait heritability. GWEBV estimation methods are categorized based on the assumptions of their prior distributions of marker effects. Genome-wide BLUP (GBLUP) methods e.g. [2], assume a normal prior distribution for all marker loci with a constant variance. In Bayesian methods, a more flexible prior distribution of SNP effects can be applied that allows for a few but with very large SNP effects whilst most are small or even zero. However, Bayesian methods often use Monte Carlo Markov chain (MCMC) algorithms which make them computationally demanding.

Meuwissen et al. [2] proposed BayesB for the estimation of SNP effects, which assumes that a fraction (1 - *π*) of the SNP have no effect and *π *SNP have an effect with a *t*-distributed prior that is more thick tailed than the normal distribution, i.e. it allows for a few SNP with very large effects and many SNP with small ones. Based on the work of [3] and [4] suggesting that normal priors give similar results as *t*-distributed priors, Luan et al. [5] used a mixture of two normal distributions as a prior, with probability *π *of the SNP effects coming from a normal distribution with a large variance and with probability (1 - *π*) from a normal distribution with a small variance. This was also justified by the observation that in practice GBLUP yields high accuracy [6], which suggests that the best predictions are obtained if the SNP with small effects are not neglected. This mixture prior distribution has two unknown parameters, *π*, the variance of the SNP with large effects. These parameters are difficult to estimate partly because the true distribution of the SNP effects is probably not a mixture of two normal distributions.

The Pareto principle, or the 80:20 rule, is often observed in economy and sociology [7]. It states that, for many events, roughly 80% of the effects come from the 20% biggest causes. This principle turned out to be widely valid in many fields, and can be generalized to (100 - *x*): *x*, where 0 *< x *≤ 50. If *x *= 50, the effects are all equal (50% of the effects cause 50% of the variance). In this study, we tried to apply this principle for the prior distribution of the SNP effects, i.e. the *x*% of the SNP with the largest effects cause (100 - *x*)% of the genetic variance (*V_g_*). Given that *π *(= *x*/100) and using the Pareto principle, the variances of the large and small SNP effects are, respectively:

(1) $$\left(\begin{array}{c} \sigma_{1}^{2}=\frac{\left(1-\pi \right)V_{g}}{\pi M}\\ \sigma_{2}^{2}=\frac{\pi V_{g}}{\left(1-\pi \right)M} \end{array}\right\}$$

where *M *is the number of SNP, such that $M\left(\pi \sigma_{1}^{2}+\left(1-\pi \right)\sigma_{2}^{2}\right)=V_{g}$. Thus, assuming the overall genetic variance is known, applying of the Pareto principle reduces the number of unknown parameters of the prior distribution from 2 to 1, i.e. *π*.

The aim of this paper is to present this novel approach using the Pareto principle applied on individual marker loci (MixP), and to compare it with other single SNP based GWEBV prediction methods in a real Norwegian Red cattle (NRF) pedigree, and on a real wheat dataset.

## Methods

### The MixP method

For the estimation of SNP effects, we used the Iterative Conditional Expectation (ICE) algorithm of Meuwissen et al. [4]. The ICE algorithm is similar to the Iterative Conditional Mode (ICM) algorithm of [8,9], except that it estimates the mean instead of the mode of the SNP effects. The latter is because the posterior of the SNP effects may be bimodal [10], in which case estimation of the mode does not make much sense (both modes may be quite far away from the mean). In order to simplify the estimation process, we will use a mixture of two normal distributions, with a 0 mean and variances $\sigma_{1}^{2}$ and $\sigma_{2}^{2}$, respectively, as a prior for the SNP effects, instead of the Spike and Slab distribution [11,12] that was used by [4].

The normal and Laplacian distributions are reported to yield similar results [9,13]. The latter is a more realistic distribution for gene effects [14]. Thus, the prior distribution of the SNP effects is assumed as a mixture of normals:

$$p\left(g_{i}\right)=\pi \phi \left(g_{i}|0,\sigma_{1}^{2}\right)+\left(1-\pi \right)\phi \left(g_{i}|0,\sigma_{2}^{2}\right)$$

where *g_i _*is the effect of SNP *i*; *π *is the probability that the SNP effect belongs to the distribution of larger variance; $\phi \left(\cdot |\mu ,\sigma_{\cdot }^{2}\right)$ is the normal distribution density function with mean *μ *and variance $\sigma_{\cdot }^{2}$. Variances of the large and small SNP effects are $\sigma_{1}^{2}$ and $\sigma_{2}^{2}$, respectively and are known from the Pareto principle (equation (1)) given that *π *is known. The model for the records is:

$$y=\mu 1+\underset{i}{\sum }b_{i}g_{i}+e$$

where **y **is a (*n *× 1) vector of *n *records, *μ *is the overall mean; **b***_i _*is a (*m *× 1) vector of the *m *'standardized' SNP genotypes, i.e., $b_{i}=\frac{-2p_{i}}{\sqrt{2p_{i}\left(1-p_{i}\right)}},\frac{1-2p_{i}}{\sqrt{2p_{i}\left(1-p_{i}\right)}},\text{or}\frac{2\left(1-p_{i}\right)}{\sqrt{2p_{i}\left(1-p_{i}\right)}}$ for SNP genotype '0 0', '0 1', or '1 1', respectively, and SNP allele frequency *p_i_*; *g_i _*is the effect of the *i*th SNP genotype; **e **is the vector of environmental effects, with $\text{Var}\left(e\right)=I\sigma_{e}^{2}$; and summation is over all SNP. The algorithm estimates the effect of every SNP in turn in an iterative manner, where the effects of all other SNP are assumed to equal their current estimates in the iteration. In iteration [*k*], let $\tilde{y_{i}}$ denote the records corrected for *μ *and for current solutions of all other SNP effects, except SNP *i*, i.e.

(2) $$\tilde{y_{i}}=y-1\mu -\underset{j\neq i}{\sum }b_{j}{ĝ}_{j}$$

The expectation of the effect of SNP *i *given $\tilde{y_{i}}$ is written out as:

(3) $$\begin{array}{c} {ĝ}_{i}=\frac{\int g_{i}\phi \left({\tilde{y}}_{i}|b_{i}g_{i},I\sigma_{e}^{2}\right)p\left(g_{i}\right)\delta g_{i}}{\int \phi \left({\tilde{y}}_{i}|b_{i}g_{i},I\sigma_{e}^{2}\right)p\left(g_{i}\right)\delta g_{i}}\\ =\frac{\pi L_{1i}{ĝ}_{1i}+\left(1-\pi \right)L_{2i}{ĝ}_{2i}}{\pi L_{1i}+\left(1-\pi \right)L_{2i}} \end{array}$$

where $L_{1i}=\int \phi \left({\tilde{y}}_{i}|b_{i}g_{i},I\sigma_{e}^{2}\right)\phi \left(g_{i}|0,\sigma_{1}^{2}\right)\delta g_{i}$ is the likelihood of the data $\tilde{y_{i}}$, i.e. L1i∝|V1i|-12 exp(-12y′˜iV1i-1yi ˜), where $V_{1i}=b_{i}{b^{\prime }}_{i}\sigma_{1}^{2}+I\sigma_{e}^{2}$. Analogously, *L*_2*i *_is defined with $\sigma_{2}^{2}$ replacing $\sigma_{1}^{2}$. In the numerator, $L_{1i}{ĝ}_{1i}=\int g_{i}\phi \left({\tilde{y}}_{i}|b_{i}g_{i},I\sigma_{e}^{2}\right)\phi \left(g_{i}|0,\sigma_{1}^{2}\right)\delta g_{i}$, where ${ĝ}_{1i}$ is the standard BLUP estimate of *g_i _*assuming that the prior variance of *g_i _*is $\sigma_{1}^{2}$. Analogously, ${ĝ}_{2i}$ is calculated assuming a prior variance of $\sigma_{2}^{2}$.

Thus, ${ĝ}_{i}$ is the weighted mean of BLUP estimates of *g_i _*assuming that the prior variance of *g_i _*is either $\sigma_{1}^{2}$ or $\sigma_{2}^{2}$ and where the weights equal the posterior probability of either belonging to the first (*πL*_1*i*_) or to the second distribution ((1 - *π*)*L*_2*i*_). As described above, the calculation of *L*_1*i *_requires the inversion of the **V**_1*i *_matrix, but the Appendix shows how to avoid this matrix inversion.

Equation (3) is applied to every SNP in turn, whilst updating the values of $\tilde{y_{i}}$ for the new solutions using Equation (2). The mean is updated by the equation:

$$\mu =\frac{1^{\prime }\left(y-\sum_{j}b_{j}{ĝ}_{j}\right)}{n}$$

Iteration is continued until the sum of the squares of the changes become less that 10^-5 ^times the sum of the squares of the solutions. We refer this method as MixP.

### Other statistical methods

Two other estimation methods were compared. BayesB is implemented as in [5], where the SNP with a small effect are assumed to come from a normal distribution with a small variance (instead of having no effect as in [2]). The variance of these small effects is sampled, and thus these small effects may be seen as modeling the polygenic background genes. As in the original BayesB, some SNP are allowed to have a big effect. The number of iterations for BayesB is 10000, and that for burn-in is 3000. The degrees of freedom for the inverse *χ*^2 ^distribution is 4.2. GBLUP is also used, as described in [2].

## Materials

### Norwegian Red cattle

The Norwegian Red cattle pedigree used in this study consisted of 19523 individuals distributed over eight generations kindly provided by GENO AS (http://www.geno.no). The data also contained an identifier indicating whether a bull was genotyped or not. A total of 2165 bulls were genotyped including 104 imported bulls. We based our simulations on this real pedigree and assumed that the genotyped animals were those in the pedigree. The cattle population data was first sorted out to make sure that the parents were placed before their offspring. The 1915 oldest genotyped bulls were marked as the training set, and the youngest 250 bulls were marked as the evaluation set, i.e. for which EBV are predicted. The total genetic variance *V_g _*and number of QTL were assumed as known parameters in this study.

### Genome structure

The parents of unknown origin were sampled from an ideal population of effective size 200 in each scenario, which was simulated for 10000 generations to achieve a mutation drift balance and linkage disequilibrium between the loci. The genome consisted of 1 Morgan/10^8 ^base-pairs. The mutation rate was 10^-8 ^per base-pair per meiosis. Markers and QTL loci were randomly selected amongst those with an allele frequency greater than 0.05. The number of markers corresponded to those inherited from the ideal population, whereas various numbers of QTL and heritabilities were simulated. QTL effects were sampled from a Laplace distribution with a 0 mean and scale parameter = 1. The genotypes of the last generation were gene-dropped into the sorted real population pedigree. No additional mutation occurs at this stage. The 1915 training bulls were genotyped and phenotyped, and the 250 evaluation bulls were only genotyped. The heritabilities, $h_{\text{chr}}^{2}$, used here should be interpreted as per chromosome heritabilities, i.e. they are about 30 times smaller than the total trait heritabilities (or reliabilities in case of daughter-yield-deviations).

### Scenarios

Three scenario parameters, i.e., chromosome heritability $\left(h_{\text{chr}}^{2}=.01,\;\cdots \;.05\right)$, marker density (*N*_mkr _= 100, 200, 500, 1000, 1500), and number of QTL per chromosome (*N*_QTL _= 5, 30, 100) varied in the simulation study. QTL were sampled randomly from the mutated loci in the ideal population simulation. Marker loci were then sampled randomly with a minimum allele frequency (MAF) of 0.05. Each scenario was repeated 100 times. The simulated genetic and environmental variances were also used to analyze the data by GBLUP, BayesB and MixP. The *π *value used for MixP was *N*_QTL_/*N*_mrk_, i.e. the ratio of number of QTL simulated to that of markers used.

### Real data analysis

The publicly available wheat dataset as described in [15] was used to test the methods on a real dataset. This dataset consisted 599 wheat lines. Grain yields in four different environments were recorded. These lines were typed at 1,447 loci. Markers with an allele frequency between 0.05 and 0.95 were used for the analysis. Ten replicates of a ten-fold cross-validation were done by randomly and evenly dividing the lines into 10 groups. In each replicate, each group was used as a validating set in turn. The correlation coefficients between GWEBV and phenotypes were averaged across all the validation folds and replicates for grain yield in each environment. The estimates of *π *for method MixP were optimized through a grid search with a step size 0.01 between 0.01 and 0.50. The heritability of grain yields used for the analysis was 0.34, 0.30, 0.41 and 0.37 for environment 1 to 4, respectively [15].

## Results

### Accuracy of GWEBV estimations

**Figure 1:**
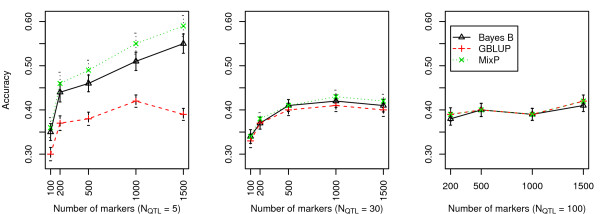
**Comparison of accuracies of BayesB, GBLUP, and MixP with a heritability per chromosome = 0.01**.

**Figure 2:**
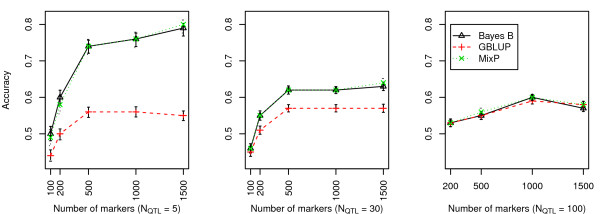
**Comparison of accuracies of BayesB, GBLUP, and MixP with a heritability per chromosome = 0.03**.

Accuracy of GWEBV estimations was measured by the correlation coefficient between GWEBV and true genotype values. Figures 1 and 2 show the accuracy of the alternative scenarios.

From Figures 1 and 2, we can see that results from MixP and BayesB were very similar and better than the GBLUP methods. A comparison between Figures 1 and 2 showed that the accuracy increased with $h_{\text{chr}}^{2}$ and *N*_mkr_. Accuracy trends on *N*_QTL _differed: the accuracy of MixP and BayesB decreased with increasing *N*_QTL_, as was found by [16]. The GBLUP method was as good as BayesB and MixP when the number of QTL is 100.

It may also be noted that the accuracy of GBLUP was very much independent of the number of QTL, which is expected since GBLUP does not give extra weight to the SNP with big effects. Hence, it does not benefit from the fact that a few genes have a big effect. This can also be seen from the formula for the accuracy of GBLUP, as shown by [17] and [18], which depends on the number of independent segments in the genome, but not on the actual number of QTL.

### Results with the wheat dataset

Table 1 summarizes the accuracies of the three methods, GBLUP, MixP and BayesB, on the wheat yields in all four available environments and shows that they are very similar. The estimates of *π*, obtained by 10-fold cross-validation, were quite high, indicating that many markers had a large effect on the traits. The curve relating prediction accuracy versus *π *was fairly flat for values of *π >*0.2 for all four yields (results not shown). Results of *π *= 0.2 for MixP are also listed in Table 1. Since *π *is not known in the wheat data, five alternative values were evaluated (0.05, 0.1, 0.2, 0.3 and 0.4) in the BayesB method. Their accuracies are very flat, i.e., no accuracy difference is greater than 0.01 within each trait. Estimates of *π *that give the top estimates for BayesB are also listed in Table 1.

**Table 1:** Accuracies of GBLUP, MixP and BayesB for the prediction of grain yield in four environments called 1-4 in wheat

Yields	1	2	3	4
GBLUP	0.53	0.50	0.39	0.46
BayesB	0.52	0.49	0.38	0.44
(*π*)	0.40	0.40	0.10	0.40
MixP	0.53	0.50	0.40	0.46
(*π*)	0.49	0.49	0.16	0.33
MixP (*π *= 0.2)	0.52	0.49	0.40	0.45

### Computational speeds

Table 2 shows the relative computational speeds, measured in CPU time of each method. With an Intel Core™Duo CPU E8500, the computational time needed for the GBLUP method is 0.14, 0.34, 0.72 and 1.13 seconds for 200, 500, 1000 and 1500 markers, respectively. Convergence was assumed for MixP and GBLUP when the sum of the squares of the deviations of the estimates of the SNP effects between subsequent iterations relative to the total sum of the squares of the estimates of the SNP effects was smaller than 10^-5^. Of all the scenarios, GBLUP is the fastest. The speed of MixP is in the same order of magnitude of that of GBLUP. BayesB is more than two orders of magnitude slower. The MixP algorithm typically converged within 50 iterations, and always converged in more than 4000 datasets.

**Table 2:** Relative computer CPU times using GBLUP, MixP and BayesB (the CPU time of GBLUP was set to 1

Number of SNP	100	200	500	1000	1500
GBLUP	1.0	1.0	1.0	1.0	1.0
MixP	1.2	1.2	1.2	1.3	1.3
BayesB	242.1	286.2	337.0	388.4	401.1

## Discussion

Here we applied the Pareto principle to estimate genome-wide EBV (GWEBV). Because a simple prior distribution was used, i.e. a mixture of normal distributions, an iterative method to calculate the BayesB type of GWEBV was obtained, which was called MixP. MixP yielded accuracies that were very similar to BayesB (Figures 1 and 2). The latter is not surprising because the prior distributions of both MixP and BayesB are mixture distributions, putting a lot of weight on a few SNP with large effects and little weight on many SNP with small effects.

For 100 or more QTL per chromosome, i.e. for more than 3000 QTL in a 30 chromosome genome with a marker density of 1500 per chromosome, there was no significant difference between MixP and GBLUP. Thus, the advantage of allowing for large SNP effects decreased when the number of QTL became large, which was also observed by Daetwyler et al. [16]. Because the estimated *π *values for grain yield were high (between 0.16 and 0.49), it appears that grain yield was also affected by many QTL, which explained the small differences in accuracy between MixP and GBLUP (Table 1). With the same wheat dataset, Crossa et al. [15] reported a slightly higher accuracy by a few percents than that found here, but this may be explained by the fact that they simultaneously fitted a polygenic effect, which was not done here. The use of the Pareto principle reduced the number of hyper-parameters, i.e. parameters of the prior distribution, from 3 (*π*, $\sigma_{1}^{2}$, and $\sigma_{g}^{2}$) to 2 (*π *and the total genetic variance *V_g_*). We will assume here that the total genetic variance was known. An estimate of *V_g _*could be obtained easily using the GBLUP model and a variance component estimation, which would provide an approximately unbiased estimate. If the total genetic variance was known, the number of parameters would be reduced to 1 (*π*), which could be estimated by cross-validation [19] in real data situations.

The reduction in number of parameters due to the use of the Pareto principle avoids the need for a multi-parameter cross-validation (for *π*, $\sigma_{1}^{2}$, and $\sigma_{2}^{2}$), which implies searching through a grid of parameter combinations. If however the multi-parameter cross validation results in a parameter combination that does not adhere to the Pareto principle and is significantly better than the best Pareto principle parameter combination, multi-parameter cross-validation should be preferred to the Pareto principle. The latter will require a very large dataset to clearly demonstrate a significant deviation of the Pareto rule.

The mean of the posterior distribution was used here to estimate SNP effects and to predict genetic values, which implemented the ICE algorithm of [4]. Alternatively, the posterior mode could have been used, which would have resulted in the ICM algorithm [8,9]. However, the use of a mixture of normals as a prior may result in a bimodal posterior distribution, see [10]. In the case of a mixture of Laplacian distributions, bimodality did indeed occur [4]. In the case of a bimodal posterior, the mode of the distribution depends on which of the two modes happens to have the higher probability density, and may thus differ widely even when the posterior distributions are quite similar. It is well established in statistics that the posterior mean maximizes the accuracy of the SNP effects and consequently yields most accurate GEBV. Since this argument did not depend on the posterior being bimodal or not, we estimated the mean of all the considered posterior distributions, as described by equation (3).

**Figure 3:**
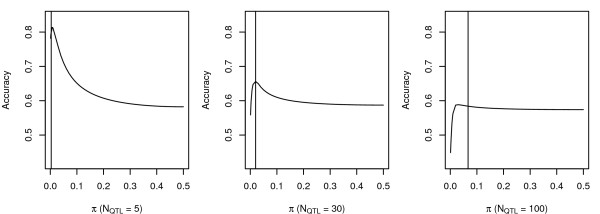
**Accuracies with different prior probabilities of SNP having a large effect on MixP with heritability per chromosome = 0.03 and 1500 markers, the vertical line indicates where *π *= *N*_QTL_/1500**.

Figure 3 shows the accuracy of GWEBV estimation with various *π*. Only the scenarios involving a number of markers of 1500 and $h_{\text{CHR}}^{2}=0.03$ were plotted. The prior *π *was used from 0.001 to 0.01 with a 0.001 step, and then to 0.50 with a 0.01 step. Accuracies were averaged over 200 repeats. The results show that unless *π *was not assumed to be much too small and the number of QTL was not small (in which case the QTL might have been mapped directly) the accuracy was not very sensitive to *π*. Similar graphs were obtained for the other scenarios (results not shown).

Alternatively, the two hyper-parameters (*π*, $\sigma_{1}^{2}$) can be estimated by MCMC estimation which 1) increases computation time significantly, and 2) may result in hyper-parameter estimates that are adapted to the current data, and will not perform well when used to predict records outside the current data, as is needed for genomic selection. An advantage of the MCMC algorithm is that the sampling also results in standard errors of the estimates. In the current algorithm, standard errors may be obtained from a parametric bootstrapping approach [20]. In this approach, replicated simulated data sets are obtained, where the estimates of the real data are to be taken as the true effects and randomly sampled error terms from the normal distribution are added to obtain simulated records. By estimating the SNP effects of the replicated data sets, the standard deviation of the SNP effects is obtained.

The Pareto principle assumes a small but non-zero effect for the SNP with small effects, whereas the original BayesB of [2] assumed no effect for such SNP. The assumption that the small SNP effects are real, implicitly implies that a polygenic effect is fitted where the marker-based relationship matrix is used to model the relationships between the animals. In the current and other simulation studies, this is usually not needed, as there are no background genetic effects next to the simulated QTL. However in real data, the fitting of a polygenic effect was found to outperform methods that did not allow for such polygenic effects [5]. In fact, in many cases, the fitting of only one polygenic effect, i.e. GBLUP, was found to yield the highest accuracy [1]. However, it may be expected that the increasing density of the SNP chips facilitates pinpointing QTL positions and methods such as BayesB and MixP will become relatively more accurate. It may also be noted that with *π *= 0.5, MixP reduces to GBLUP, i.e. optimizing the value of *π *can automatically result in GBLUP.

The simulation results presented here assumed only one chromosome, whereas most species have many chromosomes. This was however compensated for by simulating only a small (per chromosome) heritability. Using the equation for accuracy from [17] and [18], we can see that accuracies remain unchanged if the genome size and the heritability are reduced simultaneously and proportionally, i.e.:

$$r^{2}=\frac{Nh^{2}}{Nh^{2}+4N_{e}Lv}$$

where *N *is the number of training records, *L *is the genome size, 4*N_e_L *is the actual number of chromosome segments from population genetics results, *v *is the ratio of effective and actual segments, so 4*N_e_Lv *is the effective number of segments. This proportional reduction of the genome size and heritability can greatly reduce the simulation time, i.e., only around 1/30 of the computer time was needed here. Thus, using $h^{2}=h_{\text{chr}}^{2}\cdot L$, we can compare the results obtained here with other results using a full genome size.

The main advantage of MixP over BayesB is that MixP is more than two orders of magnitude faster (Table 2). This is due to the fact that BayesB requires MCMC sampling and thus many cycles in order to obtain an accurate estimate of the mean of the parameters, whereas MixP requires a limited number of iterations. Hence, MixP will be able to handle high density SNP chips such as the currently available bovine 500-800k SNP chips, whereas the MCMC based methods such as BayesB are computationally too demanding to apply to half a million or more SNP and many thousands of training records.

## Appendix

### Calculation of multivariate log-likelihood using Henderson's mixed model equations

Let us assume the following model which fits a single SNP effect:

y=bg+e

where **y **is a (*n *× 1) vector of records; **b **is a (*n *× 1) vector of standardized SNP genotypes, *g *is the marker effect of a single SNP (Var(*g*) = *σ*^2^), and **e **is a vector of error effects $\left(\text{Var}\left(e\right)=I\sigma_{e}^{2}\right)$. The log-likelihood of this model comes from the multivariate normal distribution:

$$\text{Log}L=C-.5\cdot \text{log}\left(\left|V\right|\right)-.5\cdot e^{y^{\prime }V^{-1}y}$$

where *C *is a constant and $V=\left(I\sigma_{e}^{2}+{bb}^{\prime }\sigma^{2}\right)$. The determinant of **V **can be calculated using only scalars:

$$|V|\;={\left(\sigma_{e}^{2}\right)}^{n}\left(b^{\prime }b\frac{\sigma^{2}}{\sigma_{e}^{2}}+1\right)$$

And since [21]:

$$V^{-1}=\frac{I}{\sigma^{2}}-\frac{bb^{\prime }}{\left(b^{\prime }b+\frac{\sigma^{2}}{\sigma_{1}^{2}}\right)\sigma^{2}}$$

we have:

$$y^{\prime }V^{-1}y=\sigma^{-2}\left(y^{\prime }y-y^{\prime }bĝ\right)$$

where  ĝ  is the BLUP solution of *g*:

$$ĝ=\frac{b^{\prime }y}{b^{\prime }b+\frac{\sigma^{2}}{\sigma_{1}^{2}}}$$

Thus, the calculation of **y**'**V**^-1^**y **requires only some products of vector (**y**'**y **and **y**'**b**) and the calculation of  ĝ , which was needed anyway in order to update the solutions in MixP.

## Competing interests

The authors declare that they have no competing interests.

## Authors' contributions

XY carried out the simulations, performed the analysis, and drafted the manuscript. TM designed the study and co-authored the manuscript. All authors read and approved the final version.
